# Supplementary material for: Cell Painting for cytotoxicity and mode-of-action analysis in primary human hepatocytes
Source: bioRxiv. 2025 Jan 24:2025.01.22.634152. Preprint. [Version 1] doi: 10.1101/2025.01.22.634152 (PMC11785178; doi:10.1101/2025.01.22.634152)

Supplementary materials for:

Cell Painting for cytotoxicity and mode-of-action analysis in primary human hepatocytes

Figures:

S1 - Plate effects of cell count, LDH, and MT
S2 - ToxCast cytotoxicity across cell lines and tissues
S3 - Compound number and class balance across ToxCast endpoint categories
S4 - Assay PRAUC across representation and consensus profile strategy

Figure S1. *Mean cell count (A), normalized LDH (B), and normalized MT (C) for each well position across a representative batch (prod_27).*

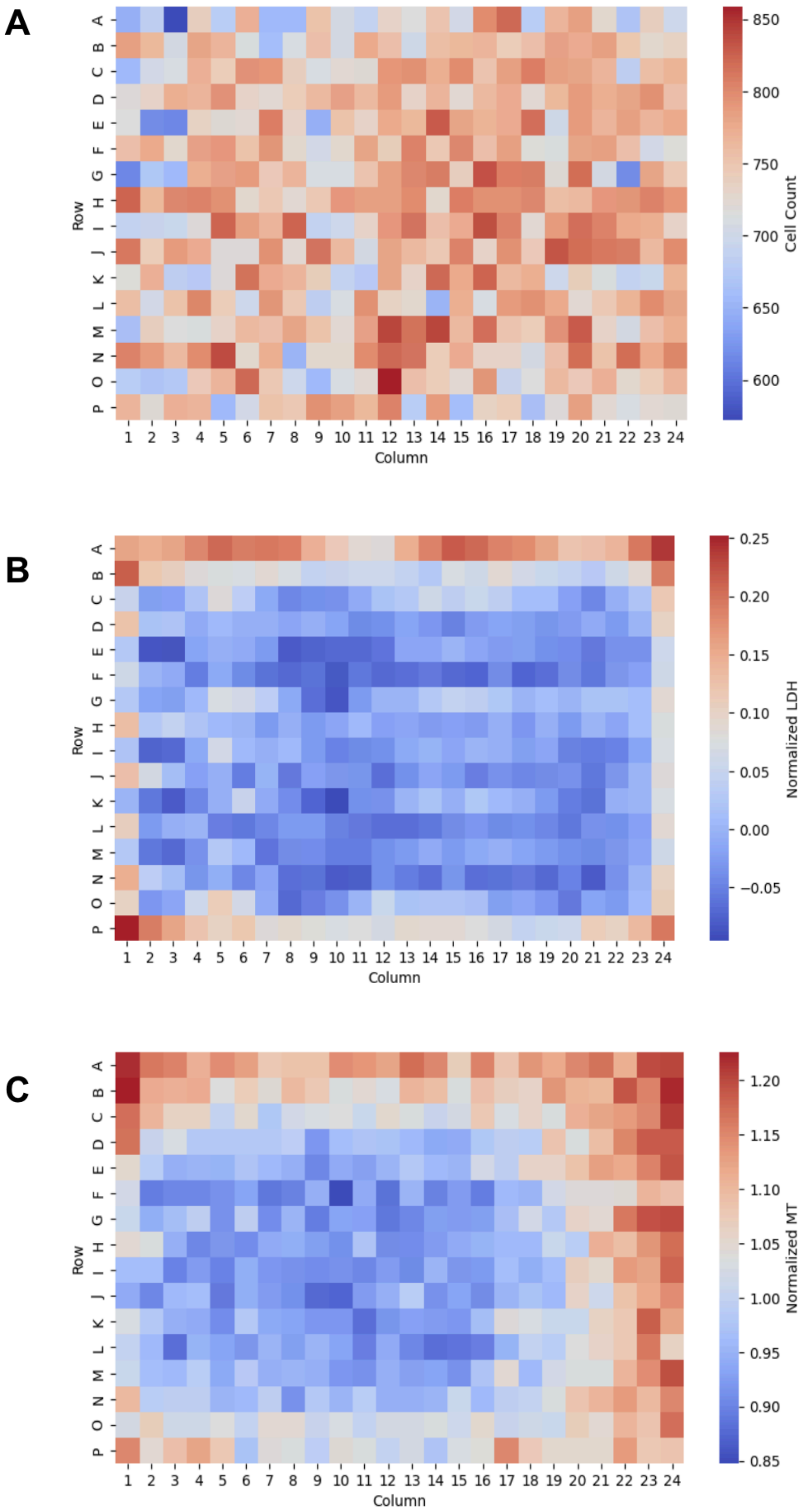

Figure S2. *Cytotoxicity AC50 values across the 12 cell lines that had >800 OASIS compounds. Rows are compounds, columns are cell lines. Compounds that either had an AC50 > 100 or that did not have a detected AC50 were set to 100 (upper range of OASIS concentrations). Lower values (darker cells) indicate greater cytotoxicity in that it occurred at a lower concentration.*

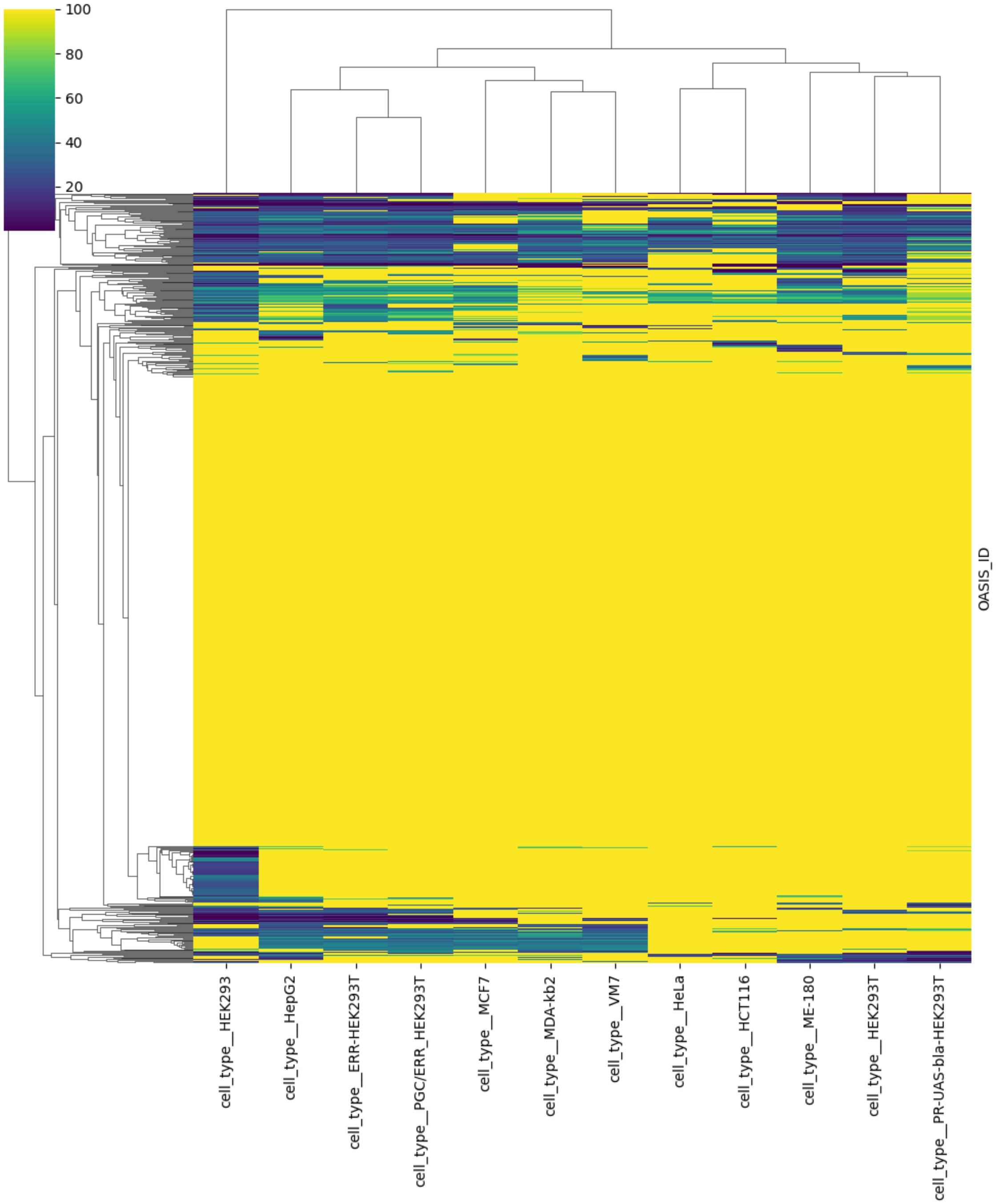

Figure S3. *Distributions of the total number of tested compounds and the percentage of positive hits for endpoints from both the cytotoxicity, cell-based and cell-free assays.*

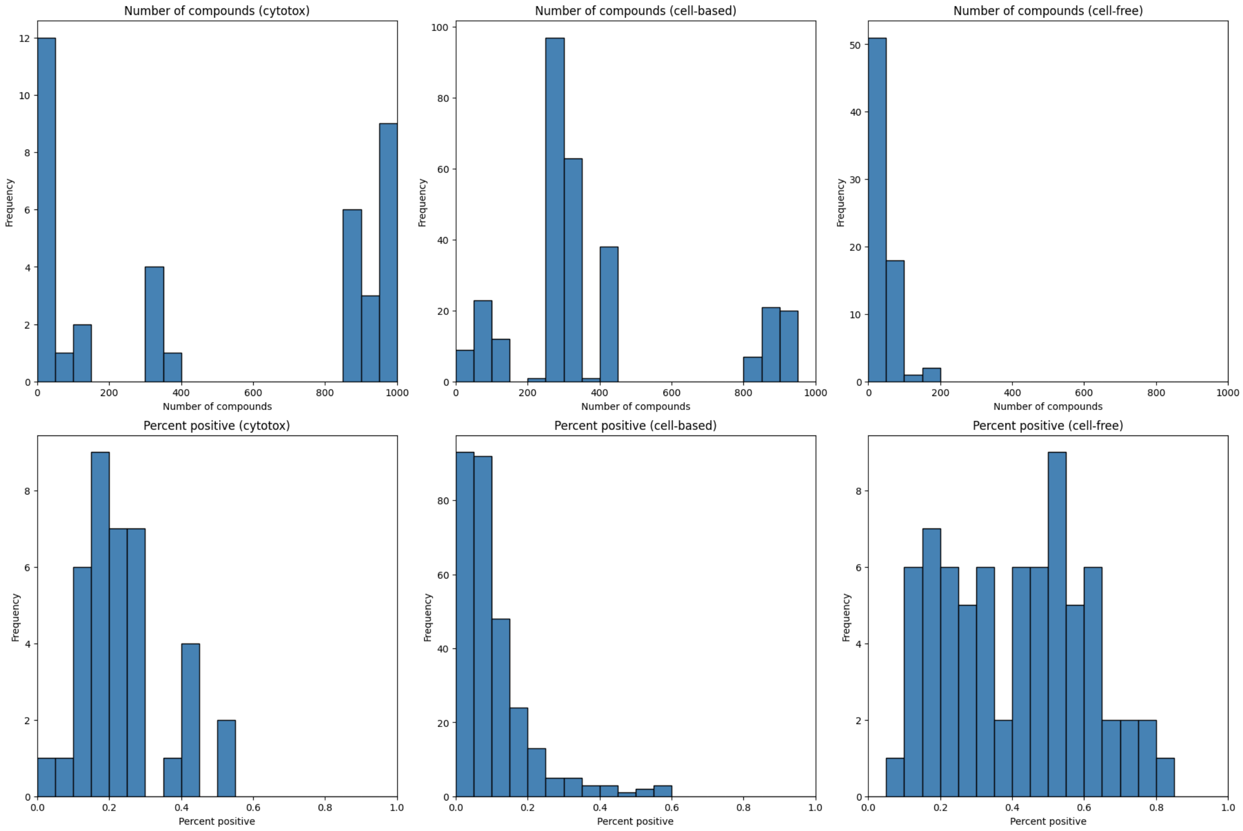

Figure S4. *Classifier PRAUC, stratified by endpoint type, across consensus profile strategy (A), and cell representation (B).*

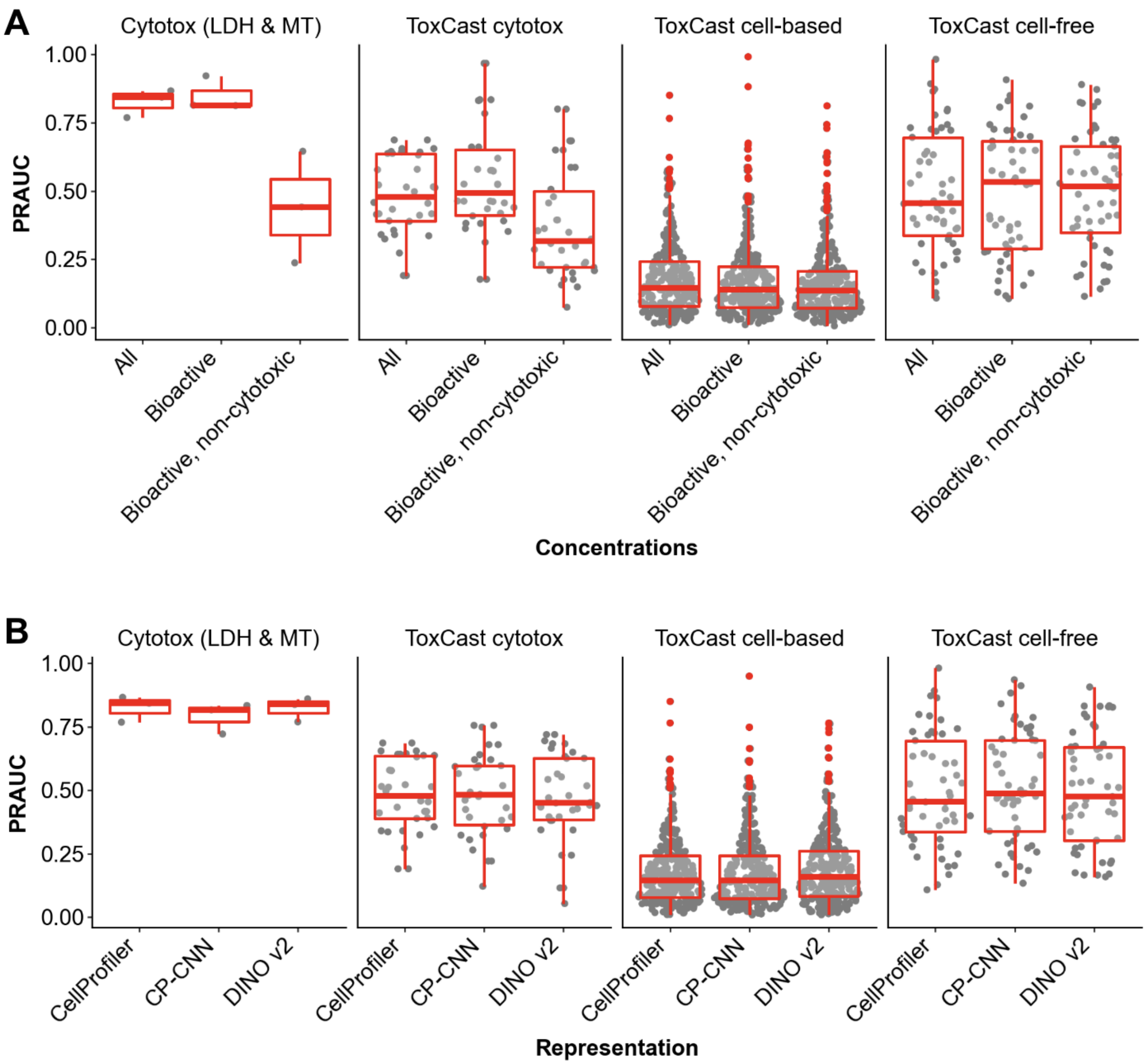

Supplement: Supplement 2 [file media-2.pdf]
